# The viscosity‐enhancing effect of carob bean gum and sodium carboxymethylcellulose when added to infant formula

**DOI:** 10.1002/fsn3.3947

**Published:** 2024-01-11

**Authors:** Kyara Baert, Mathieu Ombecq, Myriam Van Winckel, Silke Henry, Eline Tommelein, Valérie Vanhoorne

**Affiliations:** ^1^ Laboratory of Pharmaceutical Technology, Department of Pharmaceutics Ghent University Ghent Belgium; ^2^ Department of Internal Medicine and Pediatrics, Faculty of Medicine and Health Sciences Ghent University Ghent Belgium; ^3^ Department of Pharmaceutical and Pharmacological Sciences, Faculty of Medicine and Pharmacy Vrije Universiteit Brussel Jette Belgium

**Keywords:** gastro‐esophageal reflux, gastro‐esophageal reflux disease, infant formula, pediatrics, thickening agents, viscosity

## Abstract

Despite limited supporting evidence, the practice of thickening breast milk or infant formula with commercially available thickening agents is prevalent. This study explored the viscosity‐enhancing impact of carob bean gum (CBG) and sodium carboxymethylcellulose (NaCMC) when added to infant formula at various concentrations and for different thickening durations. The findings indicate that thickening leads to an exponential increase in milk viscosity, from 25% of the recommended dosage onward. This suggests that minor adjustments in dosage can significantly impact formula thickness, underscoring the importance of accurately dosing and preparing infant milk. The considerable variability in viscosity also emphasizes the need for thoughtful selection of teat size, considering the energy expenditure of the sucking infant. When using 50% of the recommended CBG dose or 25% of NaCMC, the resulting viscosity matches that of a commercially available casein‐based formula containing CBG for anti‐regurgitation. In the case of CBG, a viscosity plateau is only reached after 30 min. Therefore, educating parents on the correct handling and preparation steps for CBG‐thickened infant milk is crucial, including a 30‐min waiting period to achieve the intended thickening effect.

## Graphical abstract

Thickening infant formula with indigestible thickeners follows an exponential increase in viscosity with linearly increasing concentration. Thickening infant formula with CBG requires a 30‐min waiting period to reach final viscosity. Thickening formula with recommended doses of NaCMC provides ketchup‐like viscosity and increases sodium intake to higher levels than generally regarded as safe.

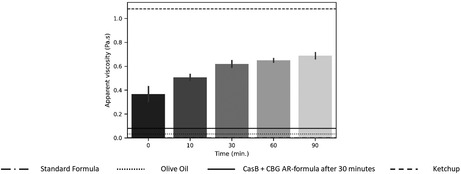

## 1 INTRODUCTION

Gastro‐esophageal reflux (GOR) is common in the first year of life for both term and preterm infants (Corvaglia, Martini, et al., 2013; Singendonk et al., 2019). Gastro‐esophageal reflux disease (GORD) develops when damage to the esophagus is expected or established. For both GOR and GORD, non‐pharmacologic (e.g., dietary management) and pharmacological measures (e.g., use of antacids or proton pump inhibitors) are applied to minimize symptoms (Corvaglia, Martini, et al., 2013; Gorsen et al., 2020; Vandenplas et al., 2005). A commonly used non‐pharmacologic intervention to manage GOR is to thicken infant milk. As such, the viscosity of the milk is increased, which slows down the flow through the teat and thus lengthens feeding time (Jadcherla et al., 2012). Additionally, when indigestible thickeners are used, the infant formula remains thick in the stomach of the infant which aims to prevent a retrograde flow of gastric content to the esophagus (Huang et al., 2002; Orenstein et al., 1987; Salvatore et al., 2018). In addition, thickened formulas are sometimes applied by speech pathologists in children with dysphagia or other difficulties with suck–swallow–breath coordination. Theoretically, the decreased flow rate of the teat provides additional time for coordinating swallowing, thereby lowering the risk of aspiration during feeding and enabling continued oral intake (Marshall et al., 2023; Pados & Mellon, 2020).

A study by Jadcherla et al. (2012) investigated different *feeding methods* and their effect on GOR in infants. The researchers concluded that faster flow rates and shorter feeding durations resulted in a higher reflux burden. It should be mentioned however that patient numbers were small, and infants did not undergo identical changes in feeding methods in this study (Jadcherla et al., 2012). Considering *thickened formulas*, early esophageal pH‐metry studies are inconclusive whether thickening effectively reduced acid burden (Bailey et al., 1987; Orenstein et al., 1987; Xinias et al., 2005). A lack of improvement in pH‐metry parameters could be explained by the fact that thickening mainly reduces postprandial reflux, which is typically nonacidic. However, two trials using esophageal pH‐impedance‐metry observed that thickening still did not result in reducing the total reflux burden when comparing feeding with and without thickening within individual patients (Corvaglia et al., 2006; Wenzl et al., 2003). Studies in which infants are randomly fed standard and thickened formula have also been performed. These studies showed that thickening does not reduce the total number of esophageal reflux episodes or symptoms but might be beneficial to parents by reducing the number of visible vomiting episodes and improving sleep (Chao & Vandenplas, 2007; Corvaglia, Spizzichino, et al., 2013; Kwok et al., 2017; Orenstein et al., 1987; Wenzl et al., 2003). Considering dysphagia, systematic reviews concluded that the overall evidence for recommending thickened liquids as an effective intervention for infants who aspirate or penetrate thin liquids is limited and of low quality (Cummings et al., 2018; Gosa et al., 2011). Results of both reviews however highlight the insufficient evidence base for this non‐pharmacological intervention and cite the need for more research to understand the efficacy and effectiveness of thickened liquid use in the pediatric population (Cummings et al., 2018; Gosa et al., 2011).

Despite the lack of evidence, thickening formula and even breastmilk is a widespread practice (Gorsen et al., 2020). Commonly used feed thickeners include digestible starch‐based thickeners made from rice or maize, and indigestible gum‐based thickeners such as carob bean gum (CBG), sodium carboxymethylcellulose (NaCMC), and xanthan gum (XG) (Kwok et al., 2017).

*Starch‐based thickening agents* are calorie‐rich, risking excessive energy intake (Gorsen et al., 2020; Khoshoo et al., 2007; Rosen et al., 2018). Starch granules swell when (pre)heated in the presence of water which is associated with loss of crystalline structures of the native starch granule and which is dependent on the amylose/amylopectin ratio of starch. A meta‐analysis by Horvath et al. indeed reported excessive weight gain in infants when rice cereal was used as thickener, probably due to a higher carbohydrate content in combination with a lower protein and fat content (Horvath et al., 2008). In addition, starch‐based thickening agents are incompatible with breastmilk due to the presence of amylase (Horvath et al., 2008). Applying starch‐based thickening agents to infant formula is therefore no longer recommended (de Almeida et al., 2011).

*CBG* is an indigestible galactomannan vegetable gum, extracted from the seeds of the carob tree. It consists mainly of high‐molecular weight hydrocolloidal polysaccharides, composed of galactose and mannose units combined through glycosidic linkages forming a galactomannan. In Europe, it is permitted for incorporation as a food additive in dietary products for infants for special medical purposes and special formulae for infants from birth onward for reduction of gastro‐esophageal reflux (REGULATION (EC), 2008). The effect of CBG on the viscosity of standard infant formula can be explained by gel formation resulting from the dissolution of mannose and galactomannans in water (Barak & Mudgil, 2014; Dos Santos et al., 2015).

*NaCMC* is a derivate of cellulose, which separates into sodium cations and an anionic cellulose backbone when it dissolves in water. The interactions between ions, the water molecule, and hydroxyl groups of the CMC molecule ensure that NaCMC‐thickened water reaches a high viscosity even at low concentrations. (Yang & Zhu, 2007) There is currently no research on the clinical effect of thickening infant formula with NaCMC, although in Europe, it is allowed for use as a food additive in dietary foods for infants for special medical purposes and special formulae for infants, as well as in dietary foods for babies and young children for special medical purposes (REGULATION (EC), 2008).

*Xanthan gum* is an extracellular polysaccharide gum produced by the microorganism *Xanthomonas campestris* and consists of glucose, mannose, and glucuronic acid (Shang & Xiong, 2010). In Europe, it is allowed for use as a food additive in dietary foods for infants for special medical purposes and special formulae for infants from birth onward in products based on amino acids or peptides with patients who have problems with impairment of the gastrointestinal tract, protein malabsorption, or inborn errors of metabolism (REGULATION (EC), 2008). Recent studies reported an increased risk of necrotizing enterocolitis (NEC) when used in infants, and its use is therefore no longer recommended (Beal et al., 2012; Patel et al., 2020; Rosen et al., 2018).

In this study, we aim to investigate the viscosity enhancing effect of CBG and NaCMC when added to infant formula at various concentrations and for different thickening durations. This way, we aim to enable a more rational selection and dosing of thickening agents for infants suffering from GOR, GORD, or dysphagia.

## 2 MATERIALS AND METHODS

### 2.1 Infant formulas, thickening agents, and reference values

A “standard” formula (Nutrilon® Profutura 1, Nutricia), commercially available in Belgium, was used to evaluate the effect of two thickening agents on milk viscosity. This formula consisted of an intact protein fraction (1.3 g/100 mL) with a 50/50 whey/casein ratio. Additionally, the formula contains 7.3 g/100 mL of digestible carbohydrates (all lactose), 0.7 g/100 mL of fibers (galacto‐oligosaccharides and fructo‐oligosaccharides), and 3.14 g/100 mL lipids. One commercially available casein‐based anti‐regurgitation (AR)‐formula, using CBG as a thickener was included as a reference (CasB+CBG AR‐formula, [Nutrilon® AR 1, Nutricia]). This AR‐formula consisted of an intact protein fraction (1.3 g/100 mL) with a 40/60 whey/casein ratio and 7.3 g/100 mL of digestible carbohydrates (all lactose). There was a fiber fraction of 0.7 g/100 mL CBG and 3.14 g/100 mL lipids.

The first thickening agent that was researched was CBG (Nutriton®, Nutricia) containing 25.1 g CBG per 100 g powder. The manufacturer's preparation instructions on the packaging dictate that the usual amount of infant formula should be spooned into a separate bowl. Next, CBG should be added to the powder and be well‐mixed to facilitate subsequent dispersion of the powder. The second thickening agent was sodium carboxymethylcellulose (NaCMC, Gelilact®, N.V. QUALIPHAR) containing 98 g NaCMC per 100 g powder. According to the manufacturer's preparation instructions, NaCMC is to be mixed with the milk powder before the milk is prepared as usual. For both products, the standard formula was adapted by adding 25%, 50%, 75%, and 100% of the dose recommended by the supplier. As a result, the evaluated concentrations for CBG included 0.85 g, 1.7 g, 2.55 g, and 3.4 g powder per 100 mL reconstituted formula while for NaCMC, 0.5 g, 1 g, 1.5 g, and 2 g powder were added per 100 mL reconstituted formula.

Standard formula without thickening, commercially available CasB+CBG AR‐formula, olive oil, and ketchup were included as viscosity reference points. They exhibited viscosities of 0.002 Pa.s (standard infant formula), 0.034 Pa.s (olive oil), 0.081 Pa.s (CasB+CBG AR‐formula), and 1.080 Pa.s (ketchup). Both the standard formula and the CasB+CBG AR‐formula samples were measured after 30 min of contact time.

### 2.2 Sample preparation

All samples were prepared per 50 mL in a 250 mL round‐bottomed flask placed in a thermostated oil bath on a magnetic stirring plate. To evaluate the effect of ascending CBG concentrations, the recommended amount of formula and CBG powder was added under continuous stirring (±300 rpm) to 50 mL of 37°C mineralized bottled water (Chaudfontaine®). Next, the sample was manually shaken for 1 min to achieve a homogenous sample, followed by continuous stirring on the thermostated oil bath for a controlled period prior to the viscosity measurements.

A different preparation protocol was used for preparation of NaCMC‐thickened preparations since a protocol similar to that of CBG led to clot formation. We opted for a preparation method that is common in daily practice. The NaCMC powder was added to 50 mL of mineralized water at 37°C, followed by 1 min of manual shaking and 9 min of magnetic stirring. This solution was then placed in the fridge for 24 h. The following day, samples were prepared by adding the recommended amount of formula under continuous stirring (±300 rpm) to 50 mL of the NaCMC dispersion, reheated to 37°C. The reconstituted sample was subsequently shaken for 1 min to achieve a homogenous sample, followed by continuous stirring on the thermostated oil bath prior to the viscosity measurements.

Commercially available CasB+CBG AR‐formula and the standard formula without thickening were prepared in the same way as CBG‐thickened formula without addition of CBG.

### 2.3 Rheological analysis

Rheological analysis was conducted 10, 30, 60, and 90 min after sample preparation. Additionally, a measurement at time point 0 min was performed for CBG‐thickened standard formula which corresponds to a measurement within a range of 3–4 min. Samples of 3.5 mL were analyzed using a stress‐controlled rotational rheometer (Haake Mars III, Thermo Fisher Scientific, Waltham, USA), with flat, polished 60 mm titanium parallel plate geometry to evaluate the viscosity. Experiments were conducted using a Peltier temperature module at a controlled temperature of 37°C. After zero gap determination at the test temperature, samples were equilibrated at the measuring gap (1 ± 0.1 mm) for 60 s prior to analysis. A rotational test with varying shear rate steps from 50 to 0.01 s^−1^ was applied in 15 equally distributed steps (logarithmically), ensuring optimal homogenization of the sample between the plates while preventing sedimentation. Each step was held for 30 s to ensure stabilization of the sample. The shear rate dependency of each formula was determined by fitting the viscosity (*η*) as function of the shear rate ($\dot{\gamma }$) with the Cross model between 0.01 and 50 s^−1^, where *k* is associated with the relaxation time and n with the power law index.

$$\eta =\frac{\eta_{0}}{1+{\left(k.\dot{\gamma }\right)}^{n}}$$

In addition, time and concentration dependency was determined by relative comparison of the viscosity between samples at the single shear rate of 50 s^−1^. This shear rate is reported as an acceptable value to simulate shear stresses of the digestive system (Cichero et al., 2011; Prakash et al., 2014). Each sample was freshly prepared in triplicate and analyzed. Both the average and standard deviation were calculated.

### 2.4 Data analysis

Results were noted using Microsoft Excel. Statistical analyses were conducted using IBM SPSS Statistics for Windows, version 29 (IBM Corp., Armonk, N.Y., USA). The within‐group measurements are assumed to be normally distributed and thus parametric statistics were applied. A one‐way between groups analysis of variance (ANOVA) with post hoc comparisons using a Tukey HSD test was conducted to explore thickening as a function of concentration or time. A *p* <.05 was considered significant.

## 3 RESULTS

**FIGURE 1:**
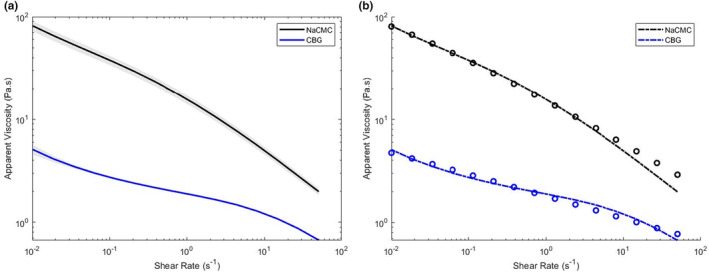
(a) Shear rate dependency of a standard infant formula, thickened with the recommended dose of CBG or NaCMC. (b) Experimental data (open circles) and fitted Cross model (dashed line) of a standard infant formula, thickened with the recommended dose of CBG or NaCMC.

Figure 1a shows the shear rate dependency of a standard formula's viscosity containing the recommended dose of CBG (i.e., 3.4 g powder containing 0.8 g CBG per 100 mL reconstituted formula) 90 min after preparation and NaCMC (i.e., 2 g powder per 100 mL reconstituted formula). Figure 1b shows the original data and fitted Cross model. The coefficient of determination is 0.989 and 0.982, respectively, for the model fit. The power law index of the fitted Cross model is 0.436 for NaCMC and 0.217 for CBG‐thickened standard formula, indicating a stronger impact of shear rate on the viscosity (i.e., more shear‐thinning) for NaCMC. In the next sections, the viscosity at a clinically relevant shear rate (50 s^−1^) is reported and compared.

**FIGURE 2:**
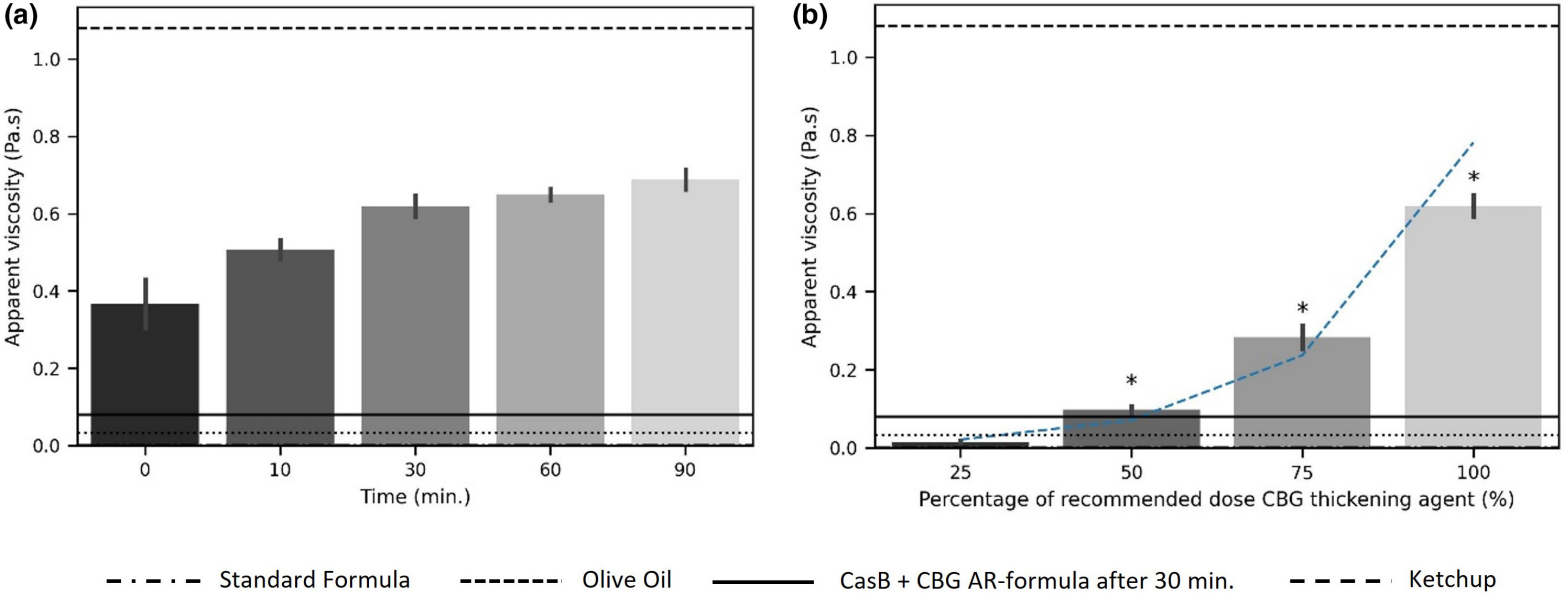
(a) Viscosity (Pa.s) of standard infant formula, thickened with the recommended dose of CBG at different time points at a single shear rate of 50 s^−1^. (b) Viscosity (Pa.s) of standard infant formula, thickened with different concentrations of CBG after 30 min at a single shear rate of 50 s^−1^. Annotated bars (*) denote which CBG concentrations generate samples with a significantly different viscosity compared to the reference CasB+CBG AR‐formula after 30 min. The fitted line represents an exponential fit to the data points with equation *y* = 0.0067e^0.0476*x*^ and *R* ^2^ of 0.9731.

Figure 2a shows the impact of contact time on the viscosity of a standard formula containing the recommended dose of CBG (i.e., 3.4 g powder containing 0.8 g CBG per 100 mL reconstituted formula). Up until 30 min, a significant increase in the viscosity of the milk was noticed, where after a plateau was reached (*p* < .01). The obtained plateau viscosity represented a sample with a consistency between olive oil and ketchup (Figure 2a). All time points showed a higher viscosity than the viscosity of the commercially available CasB+CBG AR‐formula.

Figure 2b shows the viscosity of the standard infant formula, thickened for 30 min with different concentrations of CBG. This time frame was chosen as the results from Figure 2a showed that a quasi‐stable viscosity value was reached at this time point. Except for the sample thickened with 25% CBG, all thickened samples showed a significant higher viscosity than the viscosity of the standard formula without thickening agent. The addition of 25% of the recommended CBG dose increased the viscosity, however, it did not reach the viscosity of olive oil. Addition of 50% of the recommended CBG dose generated a viscosity comparable to the commercially available CasB+CBG AR‐formula (0.086 Pa.s vs 0.081 Pa.s, respectively). Indeed, both contain identical CBG concentrations (i.e., 0.4 g CBG per 100 mL reconstituted formula). Further increase of the CBG dose exponentially amplified the viscosity in, increasing the viscosity almost by a threefold and sixfold, compared to the addition of a 50% of the recommended CBG dose for addition of 75% and 100%, respectively. Both concentrations showed significantly higher viscosities compared to the commercially available CasB+CBG AR‐formula (0.284 Pa.s and 0.620 Pa.s vs 0.081 Pa.s, respectively, both with a *p* < .001).

**FIGURE 3:**
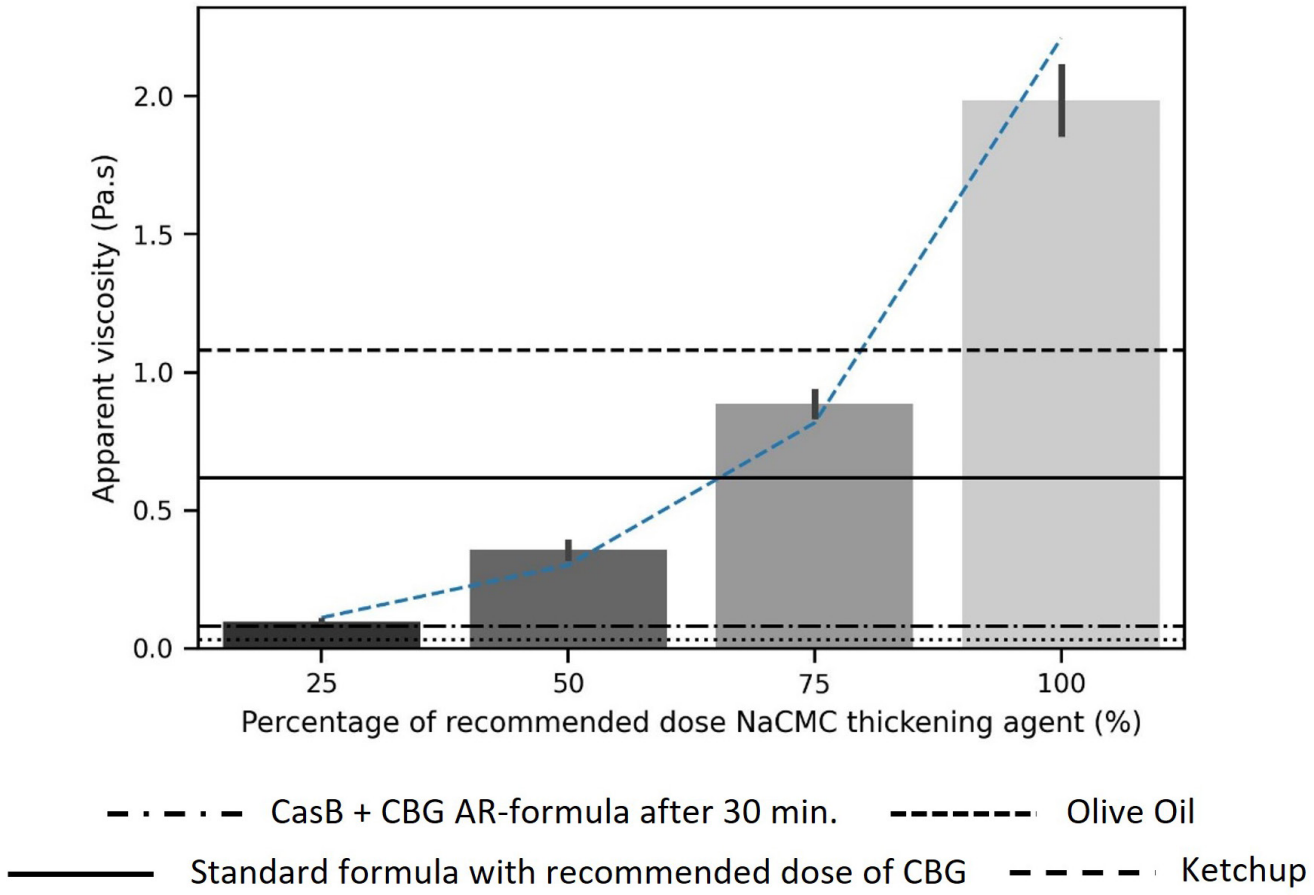
Viscosity (Pa.s) of standard infant formula, thickened with different concentrations of NaCMC. The fitted line represents an exponential fit to the data points with equation *y* = 0.0413e^0.0398*x*^ and *R* ^2^ of 0.9844. [correction added on 15 March 2024, after the first online publication: figure 3 was replaced to include the correct legend.]

Figure 3 shows the viscosity of the standard formula, thickened with different concentrations of NaCMC. The viscosity of all NaCMC samples is significantly higher than the viscosity of standard formula without thickening agent (*p* < .001). The viscosity of NaCMC‐thickened formula subsequently rises exponentially when using 25%, 50%, 75%, and 100% of the recommended NaCMC dose. Standard formula with 25% of the recommended NaCMC dose showed similar viscosity to the CasB+CBG AR‐formula (0.097 Pa.s and 0.081 Pa.s, respectively). Samples of standard formula with NaCMC concentrations of 50% of the recommended dose, showed a further increase in viscosity. Using 75% and 100% of the recommended dose of NaCMC increases the viscosity of the milk to a mean value of 0.886 Pa.s and 1.985 Pa.s, respectively, which are both significantly higher than the maximum value obtained with CBG thickening (*p* < .001).

Table 1 shows the additional daily intake of sodium when thickening is performed using different concentrations of NaCMC. An infant of 3 kg will receive a daily intake of 88 mg sodium when the lowest concentration of sodium is assumed at 25% of the recommended dose NaCMC. On the contrary, an infant of 8 kg will receive an extra daily intake of 1458 mg of sodium when the maximum regulated amount of sodium is assumed at 100% of the recommended dose NaCMC.

**TABLE 1:** Additional daily intake of sodium (mg) when thickening is performed using NaCMC when a daily intake of 150 mL milk/kg is assumed. Daily intake of sodium is given for infants from 3 kg to 8 kg body weight, using intervals of 1 kg.

Percentage of recommended dose NaCMC thickening agent	3 kg		4 kg		5 kg		6 kg		7 kg		8 kg	
	MIN^a^	MAX^b^	MIN^a^	MAX^b^	MIN^a^	MAX^b^	MIN^a^	MAX^b^	MIN^a^	MAX^b^	MIN^a^	MAX^b^
25%, i.e., 0.5 g powder	88	137	118	182	147	228	176	273	206	319	235	365
50%, i.e., 1 g powder	176	273	235	365	294	456	353	547	412	638	470	729
75%, i.e., 1.5 g powder	265	410	353	547	441	684	529	820	617	957	706	1094
100%, i.e., 2 g powder	333	547	470	729	558	911	706	1094	823	1276	941	1458

^a^ Minimum amount of sodium in NaCMC according to a 1:1 substitution degree (8%).

^b^ Maximum amount of sodium in NaCMC according to EU No 231/2012 (12.4%) (EFSA, 2018).

## 4 DISCUSSION

This study reports on the influence of indigestible thickeners on the viscosity of a standard infant formula. In terms of shear rate dependency, the viscosity of NaCMC‐thickened infant formula shows greater variation than CBG‐thickened formula given the higher power law index. As smaller teat sizes will result in higher shear values, it can be hypothesized that the viscosity of NaCMC‐thickened formula can be more affected by the teat size. It is however unclear how this rheological behavior might affect feeding and teat size selection in practice. Both CBG and NaCMC are capable of thickening standard infant formula, already from 25% of the recommended dosage onward. For both thickening agents, the viscosity increasing effect displayed an exponential dose dependency, with NaCMC being capable of reaching higher viscosities than CBG within their recommended dosages. As a result, a wide variety of viscosities can be obtained depending on the used dosage. It is important to note that for CBG, a thickening plateau phase was only reached after 30 min of contact time, which should be communicated when preparing thickened infant milk.

Despite limited evidence about the effectiveness of thickened standard infant formula to reduce GOR episodes (Corvaglia et al., 2006; Wenzl et al., 2003) or in children with dysphagia (Cummings et al., 2018; Gosa et al., 2011), thickening of infant feeding is included in de NASPHGAN and ESPHGAN recommendations for the treatment of GORD (symptoms) (Rosen et al., 2018) and is observed to be a widespread practice (Gorsen et al., 2020). Nevertheless, there is very little research about which thickening agent at what concentration is to be preferred, as well as which preparing methods should be recommended (Horvath et al., 2008; Kwok et al., 2017).

### 4.1 Preferred thickening agent

As previously mentioned, XG and starch are not preferred due to an increased risk of NEC for XG (Beal et al., 2012; Patel et al., 2020; Rosen et al., 2018) and due to the risk of excessive energy intake and incompatibility with breastmilk for starch (de Almeida et al., 2011). The two remaining gum‐based thickeners, CBG and NaCMC are not susceptible to amylase in breastmilk and do not (or barely) increase the calorie level of the milk (de Almeida et al., 2011). Additionally, for CBG, the reached viscosity in the bottle remains stable when coming into contact with gastric content as it is unaffected by pH and temperature (Alves et al., 1999), which might not be the case for NaCMC, as there is one study suggesting that the viscosity of a NaCMC water solution decreases with lowering pH (Yang & Zhu, 2007). Considering the clinical effectiveness of thickening agents to reduce regurgitation or reflux, a meta‐analysis summarizing four trials using commercially available AR‐formulas thickened with CBG, showed that CBG tended to lower the amount of regurgitation periods per day along a significant increase in the number of asymptomatic infants after the trial period (Hegar et al., 2008; Iacono et al., 2002; Moya et al., 1999; Vandenplas et al., 1994). However, no clinical trials investigated the effects of adding CBG to standard formula. There is however no scientific reason to believe that the effect of added CBG would be less than when the thickening agent was previously added by the manufacturer, subject to correct preparation. No in vivo studies using NaCMC for the reduction of regurgitation were published. Indeed, in 2018, the European Food Safety Authority released a call to address this lack of data about NaCMC for use in food for young infants (EFSA, 2018). In addition, thickening with NaCMC largely contributes to the daily sodium intake as can be seen in Table 1. According to the European Food Safety Authority, safe sodium intakes are 1100 mg/day for children aged 1–3 years. For infants aged 7–11 months, an intake of 200 mg/day is generally accepted as safe (U.S. Department of Agriculture A, 2020; EFSA, 2019). Taking this into account, together with the results of this study, one could conclude that CBG should be the first‐choice thickening agent, provided attention is paid to correct preparation methods.

### 4.2 Preferred concentrations

This study shows that a broad range of viscosities can be reached by adding CBG or NaCMC to a standard infant formula. It was shown previously that commercially available AR‐formulas with a CBG concentration ranging from 0.33 g/100 mL to 0.45 g/100 mL can reduce the number of regurgitation episodes in infants (Georgieva et al., 2016; Miyazawa et al., 2004). Whether a higher concentration leads to a more prominent reduction of regurgitation episodes is conflicting (Georgieva et al., 2016; Miyazawa et al., 2004). This analysis revealed that adding 50% of the recommended CBG dose (0.4 g/100 mL) to a standard formula resulted in a similar viscosity as a commercially available CasB+CBG AR‐formula. Concerning NaCMC, a similar viscosity is already reached when only 25% of the recommended dose is used. One can conclude that this should be first choice concentrations. Nevertheless, when the effect seems insufficient, higher concentrations of thickening agents might be considered. However, possible consequences should also be thought of.

*First*, extensive thickening is associated with more difficulty to suck the formula through the teat (Miyazawa et al., 2004). Indeed, this research shows that higher concentrations of CBG and NaCMC increase the viscosity in a nonlinear way. As a result, very high viscosities can be reached, even with minor adaptations. Furthermore, the viscosity of NaCMC‐thickened standard formula with recommended dose showed an extremely high viscosity (1.98533 Pa.s). Indeed, low concentrations of NaCMC are known for causing already high viscosities (Yang & Zhu, 2007). The viscosity of NaCMC‐thickened formula with the recommended dose is at least twice the value of CBG‐thickened formula. Therefore, the recommended dose of NaCMC is questionable and this viscosity increase should be taken into account when selecting teats of the feeding bottle for compatibility. Indeed, a recent study showed that high viscosities often caused blockage of a test syringe, which can also occur using teats (Marshall et al., 2023). Moreover, it raises concerns about the energy expenditure of an infant trying to suck this thickened formula through the teats. *Second*, both investigated thickening agents are indigestible fibers and it was shown that all indigestible thickeners can cause gastrointestinal symptoms like abdominal pain, diarrhea, and constipation (Kwok et al., 2017). A gradual increase in added dose of CBG‐based thickening agent to infant feeding over several days could reduce the risk of cramps as it gives the time to the gastrointestinal system to adjust (Georgieva et al., 2016; Hegar et al., 2008). *Finally*, nutritional aspects should be taken in consideration since CBG was suggested to affect the intestinal absorptions of minerals like calcium, iron, and zinc more than digestible carbohydrates (Bosscher et al., 2003; Corvaglia, Martini, et al., 2013; Hegar et al., 2008). This suggestion has though never been confirmed in in vivo studies since no trials formally assessed failure to thrive or micronutrient deficiencies as an outcome. It should nevertheless be mentioned that commercially available AR‐formulae are designed to provide a micronutrient density and osmolality appropriate to the needs of infants, which might be affected when thickeners are added to formula feeds (Kwok et al., 2017).

### 4.3 Preparing methods

The finding that thickening with CBG continued up until 30 min after adding CBG, followed by a stabilization, is important for current advice regarding the use of CBG. These results confirm previous findings where infant formulas with added gum‐ and starch‐based thickening agents were significantly thicker after 30 min compared with 5 min of stand time (Gosa & Dodrill, 2017). Additionally, Koo et al reported a stabilization of viscosity of all gum‐based thickeners, such as CBG, prior to 120 min (Koo et al., 2019). For NaCMC, substantial clotting was observed when the recommended preparation method by the supplier (i.e., adding dry powder to powder formula after which water is added) was utilized. The presence of these clots and undissolved particles impeded correct rheological analysis, and subsequently, the preparation method had to be altered. This way, a homogenous dispersion was generated. In practice, parents applying the method as suggested by the manufacturer, might need to extensively shake the bottle to disperse the clots. Extensive shaking can generate air bubbles in the milk, which leads to an aggravation of the reflux episodes as the infants consummate more air. This study shows that attention should be paid to explaining correct preparation methods when the use of indigestible thickeners is advised.

### 4.4 Strengths and limitations

This study's strengths include the use of a rheometer as it is the recommended and user‐independent method for assessing thickened fluid properties. However, the absence of specific viscosity data for therapeutic effectiveness in GORD and dysphagia limits comparability with therapeutic standards. Additionally, our investigation into the impact of the addition of NaCMC on a standard formula is the first of its kind. Although the use of NaCMC as thickening agents is widely spread as a strategy in the treatment of GORD, no clinical information is available. This study provides more information about the degree to which NaCMC thickens the standard formula, giving relevant implications for practice and answering the call of the European Food Safety Authority to address this lack of data about NaCMC (EFSA, 2018).

A first limitation is the use of commercially available (AR‐)formulas, as the composition of these formulas may vary in different countries, although the same brand name is used. As a result, this study might be difficult to extrapolate to other countries as different compositions could influence the results. Indeed, even formulas with similar ingredients seem to present different thickening patterns, which warrants a more in‐depth exploration of this phenomenon (Marshall et al., 2023). A second limitation is the inability to obtain the initial time point (0 min) for the standard formula with CBG thickener due to practical reasons. The actual measurement at time point 0 represents a measurement within 3–4 min after 1 min of shaking the sample, introducing measurement inaccuracies. Finally, while shear rate's impact on viscosity was examined, thixotropy (time‐dependent shear thinning) was not investigated, suggesting a potential area for future research. However, selection of clinically relevant settings to study thixotropy of the thickeners remains challenging.

### 4.5 Implications for practice and research

It is important to mention that thickening infant formula should only be recommended as a last resort in the management of infant feeding. Other strategies to support reducing teat flow rate (e.g., external pacing, employing a slower flowing teat) should ideally be considered prior to the use of thickened infant formula. However, when thickening formula is desirable, CBG might be the preferred agent due to its no‐calorie properties, compatibility with all liquids, and—compared to the other thickening agents—its easier and from a microbiological point‐of‐view also potentially safer preparation method. One should however consider that high viscosities can be reached with lower concentrations than mentioned on the packaging. Likewise, it is important to note that the addition of indigestible thickeners increases the viscosity exponentially, meaning that slight increases in concentrations can dramatically influence the thickness of the formula. Measuring the viscosity of CBG‐thickened formula at different time points allowed the estimation of time needed to reach an optimal viscosity which appeared to be 30 min. This finding can be translated to clinical practice and should formally be included in future recommendation guidelines. Parents should therefore be informed about correct handling and preparation as thickening with CBG requires a 30‐min waiting period to reach final viscosity. This implicates that the bottle must be prepared 30 min before consummation by the infant, which is higher than the 10 min, recommended in other research (Marshall et al., 2023).

Both CBG and NaCMC dosed as recommended by the supplier thicken the milk to viscosities, even sometimes exceeding that of the ketchup reference. Consequently, the teat size should be chosen with careful consideration as feeding bottles are often not designed for thickened formulas (Cichero et al., 2013). When the teat size is too small, infants tend to be exhausted from trying to suck the thickened formula from the bottle. When the teat size is too large, there is a risk of aspiration with resulting respiratory problems (Gosa & Choquette, 2021).

In this study, the effect of acidic environment on the viscosity of thickened formulas was not taken into consideration. This could however be interesting, as some thickening agents, such as NaCMC, and proteins could be affected by different pH conditions. Future research is needed to investigate the effect of pH on the viscosity. Furthermore, a simulation of the gastric content by addition of pepsin and/or using a dynamic model of the stomach and small intestine (Salas‐Bringas et al., 2015) could be interesting as well. Future investigations are needed to determine an exact level of viscosity with clinical impact on the treatment of GORD or dysphagia. Larger controlled trials are needed to investigate the efficacy and safety of thickening agents.

## 5 CONCLUSION

Thickening infant formula with CBG and NaCMC follows an exponential increase in viscosity meaning that slight increases in concentrations can lead to a large increase in thickness. Thickening using CBG and NaCMC at only 50% and 25%, respectively, of their recommended dose resulted in similar viscosities as a commercially available CasB+CBG AR‐formula and might be sufficient to reach clinical goals. Health‐care providers should give elaborate advice concerning the use of thickening agents such as precise dosing, prolonged stand time before consummation, and corresponding teat size selection.

## AUTHOR CONTRIBUTIONS

**Kyara Baert:** Formal analysis (equal); investigation (equal); methodology (equal); writing – original draft (equal). **Mathieu Ombecq:** Formal analysis (equal); investigation (equal); methodology (equal); writing – original draft (equal). **Myriam Van Winckel:** Conceptualization (supporting); writing – review and editing (supporting). **Silke Henry:** Conceptualization (equal); formal analysis (equal); methodology (equal); supervision (equal); writing – review and editing (equal). **Eline Tommelein:** Conceptualization (equal); formal analysis (equal); methodology (equal); resources (equal); supervision (equal); writing – review and editing (equal). **Valérie Vanhoorne:** Conceptualization (equal); formal analysis (equal); investigation (equal); methodology (equal); resources (equal); supervision (equal); writing – review and editing (equal).

## FUNDING INFORMATION

The authors declare that no funds, grants, or other support were received during the preparation of this article.

## CONFLICT OF INTEREST STATEMENT

The authors have no relevant financial or nonfinancial interests to disclose.

## ETHICS STATEMENT

This study does not involve any human or animal testing.

## Data Availability

The datasets during and/or analyzed during this study available from the corresponding author on reasonable request.
